# Impact of Temporary Nitrogen Deprivation on Tomato Leaf Phenolics

**DOI:** 10.3390/ijms12117971

**Published:** 2011-11-16

**Authors:** Camille Bénard, Frédéric Bourgaud, Hélène Gautier

**Affiliations:** 1 INRA UR 1115 Horticultural *Plants* and Culture *Systems* (*PSH*), Domaine Saint Paul, F-84914 Avignon, France; E-Mail: camille.benard@avignon.inra.fr; 2 UMR 1121 University of Lorraine (INPL)-INRA Agronomy and Environment Nancy-Colmar, ENSAIA 2, avenue de la forêt de Haye F-54505 Vandoeuvre-lès-Nancy, France; E-Mail: frederic.bourgaud@ensaia.inpl-nancy.fr

**Keywords:** chlorogenic acid, rutin, nitrogen deprivation, phenolic compound, tomato

## Abstract

Reducing the use of pesticides represents a major challenge of modern agriculture. Plants synthesize secondary metabolites such as polyphenols that participate in the resistance to parasites. The aim of this study was to test: (1) the impact of nitrogen deficiency on tomato (*Solanum lycopersicum*) leaf composition and more particularly on two phenolic molecules (chlorogenic acid and rutin) as well as on the general plant biomass; and (2) whether this effect continued after a return to normal nitrogen nutrition. Our results showed that plants deprived of nitrogen for 10 or 19 days contained higher levels of chlorogenic acid and rutin than control plants. In addition, this difference persisted when the plants were once again cultivated on a nitrogen-rich medium. These findings offer interesting perspectives on the use of a short period of deprivation to modulate the levels of compounds of interest in a plant.

## 1. Introduction

During their development, plants produce different vital metabolites that are commonly referred to as primary metabolites. They also synthesize numerous compounds qualified as “secondary”, the functions of which have not always been identified yet but which remain fundamental, notably regarding the adaptation of plants to their environment. Of these secondary compounds, polyphenols are, amongst others, involved in the mechanisms of resistance to biotic and abiotic stresses. In tomato, leaves are a site of infection by parasites, and the damage caused can have dramatic issues on plant productivity, reducing plant photosynthesis and inducing a loss of fruit yield. Chlorogenic acid and rutin are the principal phenolic compounds found in tomato leaves [1]. It has been shown that these compounds intervene directly in resistance to fungal and bacterial pathogens [2–4]. However, their protective role against herbivores remains controversial [5–7].

A reduction in the use of pesticides represents a major challenge for modern agriculture, particularly in the context of preserving the environment and human health. For this reason, improving the resistance of plants to parasite attacks by modulating their cultivation conditions offers interesting opportunities [8]. In particular, a deficit in mineral elements has often been employed to study the regulation and formation of secondary metabolites, including phenolic compounds [9,10]. Several studies showed that a reduction in nitrogen nutrition increases the levels of phenolic compounds in leaves [1,11–14]. Nitrogen deprivation also induces negative effects on plant growth [15,16] and yield [17]. It would therefore be interesting to determine cultivation methods that could improve plant resistance without affecting production; for example by subjecting plants to moderate stress or by alternating periods of environmental restriction with periods of optimum conditions. This latter strategy suggests knowing whether these modifications to secondary metabolism will persist once the environmental restrictions have been lifted, allowing the plant to increase its resistance to other types of biotic or abiotic stress.

Such responses had already been observed in the tomato and grasses when they were subjected to successive periods of water stress [18,19]. These studies showed that moderate water stress induced an acclimatization of plants that allowed them subsequently to better tolerate more severe water stress. On the other hand, following a period of nitrogen deficit, Scheible and co-workers [20] showed different evolution pattern of phenylpropanoïd metabolites in *Arabidopsis* plantlets replaced on a nitrogen-rich substrate. The behavior of tomato plants following a return to non-limiting conditions after an episode of nitrogen deprivation has not been studied. The aim of the present study was therefore to determine the strategy of this plant in the event of temporary nitrogen deprivation followed by a return to a nitrogen-rich substrate. Biomass growth, tissue contents in carbon and nitrogen and the accumulation of phenolic compounds were thus determined following a period of deprivation and five days after a return to normal conditions.

## 2. Results

### 2.1. Effects on Plant Biomass

**Figure 1:**
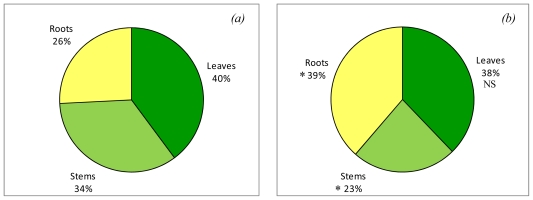
Percentage distribution of fresh biomass produced between the three plant compartments: roots, stem and leaves. Plants belong to the experiment A, and were harvested at the end of the period of N deprivation (Harvest 1, Figure 2). (a) Plants grown for 10 days on a rich medium (7 mM NO_3_ ^−^) or (b) plants grown for 10 days on nitrogen deficient medium (0 mM NO_3_ ^−^). Stars (*) indicate significant difference between the two treatments, *i.e.*, plants grown on 7 or 0 mM; NS, not significant.

The distribution of plant biomass was modified by the period of deprivation (Figure 1). The proportion of fresh matter allocated to the roots was significantly greater in plants growing under nitrogen deprivation (39%) than in plants growing on the rich medium (26%). This increase in the fresh matter allocated to the roots was achieved mainly to the detriment of the stems, as the fresh matter assigned to leaves was only slightly modified. Similar results were observed during experiment B, when plants were subjected to a longer period of deprivation (results not presented).

At the end of the experimental period, a reduction of approximately 50% in total biomass was observed in treated plants (Figure 3). After a return to the rich medium for five days, the growth of plants that had been nitrogen-deprived was not sufficient to make up for their delayed development. After five days of a return to 7 mM, the reduction in fresh matter in previously treated plants compared to controlled plants, ranged from 12%–31% in the roots, 59%–61% in the stems and 68%–73% in the leaves, the longer period of nitrogen deprivation during experiment B inducing a more marked reduction in biomass.

### 2.2. Effects on Plant Composition

Nitrogen deprivation induced a marked reduction in the percentage of total nitrogen in leaves that reached 62% and 71%, respectively, during experiments A and B (Figure 4). After a return to a rich medium, the percentage variations were reduced to 21% and 18%, respectively, thus demonstrating the very rapid nitrogen uptake dynamic of tissues. However, the periods of deprivation and the return to a rich medium did not affect tissue carbon contents.

After the period of deprivation, chlorogenic acid levels were more than doubled in treated plants when compared with control plants (increase of 136% and 117% during experiments A and B, respectively; Figure 4). Rutin levels in treated plants did not differ from those in control plants during experiment A (increase of 7%), but were doubled in treated plants versus control plants in experiment B (increase of 119%). These differences were further amplified after a return to a rich medium. Indeed, the chlorogenic acid contents found during both experiments continued to rise after a return to a rich medium (increase of 194% and 340% in experiments A and B, respectively). This trend was also observed for rutin, levels of which increased by 50% and 110% respectively in experiments A and B.

## 3. Discussion

### 3.1. Effect of Nitrogen Deprivation on Plant Development

Within a ten day period of nitrogen deprivation, the plants had developed a strategy for fresh matter distribution, with preferential growth of its root system biomass. This development strategy is frequently observed [21,22] because a plant will make every effort to explore its cultivation medium to find the elements it is missing. Generally speaking, the production of fresh matter was almost reduced by half in treated plants, indicating that the deprivation duration was long enough to induce morphological modifications. The return to a rich medium for five days induced a resumption of growth but it was not enough to make up for the delay suffered during this period.

### 3.2. Effect of Nitrogen Deprivation on Leaf Composition

Although the tomato plants contained quite high levels of nitrogen (accumulated during the 10-day acclimatization period on a 7 mM NO_3_ ^−^ medium), after just a few days of deprivation both their nitrogen status and metabolism were affected. Indeed we observed a very marked reduction in the percentage of nitrogen in tissues after the period of deprivation (6 g N per 100 g dry matter in plants remaining at 7 mM and approximately 2 g N per 100 g dry matter in treated plants), and an increase in the content of secondary metabolites (Figure 4). These observations confirmed the findings of previous studies using polyphenol concentrations as an indicator of plant nitrogen status [23].

During the period of deprivation, a modification to the nitrogen supply from the nutrient solution was accompanied by changes to secondary metabolism in the leaves. Levels of chlorogenic acid were doubled (increase of 136% and 117%) in treated plants. Similar variations had previously been observed in *Arabidopsis* plantlets subjected to two days of deprivation [20], tomato plants subjected to 4 or 8 days of deprivation [14] and camomile plants subjected to 12 days of deprivation [24].

Scheible and co-workers [20] observed a marked rise in rutin concentrations, but in general the variations were smaller regarding the content in compounds synthesized at the start of, or midway through, the polyphenol biosynthetic pathway, such as hydroxycinnamic derivatives. During the present study, we also observed an increase in rutin levels during experiment B, but it was the concentrations of chlorogenic acid, a hydroxycinnamic derivative occurring midway through the biosynthetic pathway, that were particularly affected. This difference in the type of compounds that accumulated may have been linked to the plants under study. Indeed, in *Arabidopsis*, hydroxycinnamic derivatives such as chlorogenic acid do not accumulate in the tissues, unlike what happens in the tomato [2,14].

The aim of this study was to determine if, after a return to more favourable cultivation conditions (7 mM NO_3_ ^−^), a plant retained any metabolic stigma of a period of deprivation. During a previous study, a supply of NO_3_ ^−^ to nitrogen-deprived *Arabidopsis* plantlets (at cotyledon stage) had caused a repression of genes involved in polyphenol synthesis that was detectable after less than 30 minutes and marked after 3 hours [20]. In addition, compounds whose synthesis had been stimulated by deprivation (cafeic acid, ferulic acid and rutin) remained at higher levels between 3 and 24 hours after the addition of nitrogen when compared with control, non-stressed plants. The results presented here confirm that even over a longer period of a return to a rich medium (five days) and in more developed plants (2 leaves tomato plants at the beginning of the experiment), the effects of stress remained visible: the levels of chlorogenic acid and rutin were higher in plants that had previously been deprived when compared with control plants.

Moreover, the content in rutin and chlorogenic acid in the treated plants strongly increased after a return to the rich medium compared to the control. To the best of our knowledge nothing has been established on how these compounds are catabolised or metabolised after their synthesis. So it may be possible that the phenolics previously synthesized remain in the plant for such a period. In addition, the recovery of nitrogen (as shown by increased leaf nitrogen content) may have permitted a strong enhancement of phenolics biosynthesis. Indeed, C-based secondary metabolites do not contain N, but the metabolic machinery necessary for their synthesis does need and does contain N [12]. Using the Growth Differentiation Balance Hypothesis [25], we may hypothesise that after the return to the rich medium, treated plants are still subjected to growth restriction but no more to photosynthesis limitation (see Figure 1 in [12]), so that more carbon is available for the synthesis of C-based compound such as phenolics.

Results presented here demonstrated that the accumulation of secondary metabolites persisted after a temporary nitrogen depletion, and consequently growing plants under non optimal conditions using temporary environmental restriction offer optimistic prospects for increasing plant resistance. Moreover it is possible to draw some parallels between data presented herein and what is already known regarding the response of secondary metabolism to biotic stress [4,26,27]. The defense mechanisms of plants against pathogens trigger a succession of phenomena. After recognizing the pathogen, a local response develops (hypersensitive response), followed by a global response at the scale of the whole plant (acquired systemic response) which endows it with long-term protection against future attacks. It is thus possible to imagine a similar behavior in plants when faced with an abiotic stress such as a deficit in mineral elements. For example, mechanisms such as enzyme synthesis, developing during an initial episode of stress, may induce a more intense response, *i.e.*, an elevation of polyphenol levels that becomes more marked during a subsequent episode of deprivation. It would therefore be interesting to quantify polyphenol levels in a plant following several episodes of nitrogen deprivation.

## 4. Experimental Section

### 4.1. Plant Materials, Growth Conditions and Nitrogen Treatments

Tomato plants (*Solanum lycopersicum* cv Microtom) were grown under hydroponic conditions in a phytotron. The temperature in the growth chamber was 23–25 °C during the day and 18–20 °C at night, with relative humidity of 75% during the day and 70% at night. The mean photosynthetically active radiation (PAR) was 250 μmol m^−2^ s^−1^ and the photoperiod was 14 hours. Three weeks after germination in potting soil irrigated with water, plants with two pairs of leaves and 2 cm of roots were transferred to a container-based hydroponic cultivation system (1 plant/liter). The nutrient solution was continually aerated by a bubbling system and was renewed every three days (Tables 1 and 2). Two experiments, A and B, were carried out.

**Figure 2:**
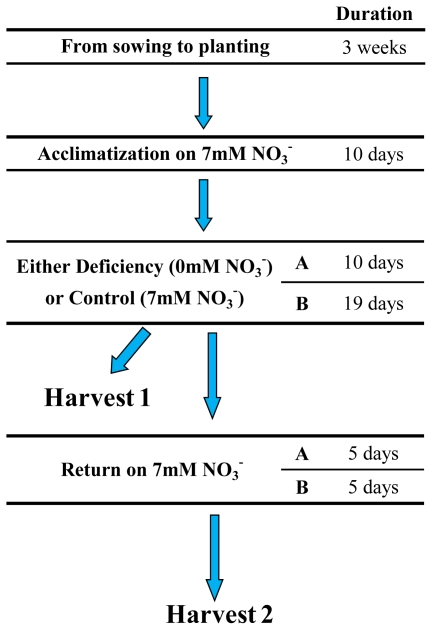
Simplified presentation of the experimental protocol used to study the effect of temporary nitrogen deprivation in tomato. During the two experiments, after 10 days of acclimatization at 7 mM NO_3_ ^−^, 8 plants were placed in a situation of deprivation (treated plants at 0 mM NO_3_ ^−^) and 8 control plants were placed on a rich medium containing 7 mM. 4 treated plants and 4 control plants were harvested following the period of deprivation (Harvest 1: H1). The four remaining plants per treatment were cultivated on the rich medium for a further 5 days before being harvested (Harvest 2: H2). During experiment A, the plants were deprived for 10 days, and during experiment B, for 19 days.

**Figure 3:**
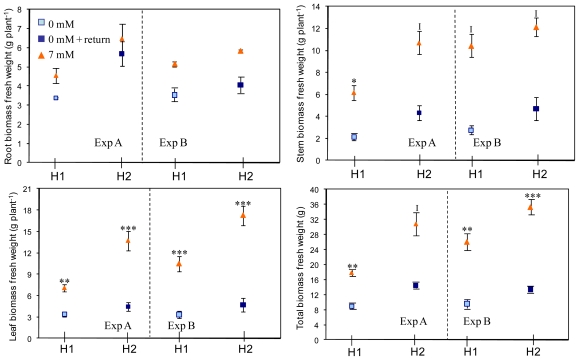
Evolution of fresh weight in leaves, stems, roots and total biomass during experiments A (Harvest 1 at 10 and Harvest 2 at 15 days) and B (H1 at 19 and H2 at 24 days). Data are mean ± SE calculated on four plants, and expressed as g per plant. Stars indicate significant effect of nitrogen. *, *P* < 0.05; **, *P* < 0.01; ***, *P* < 0.001; I, interaction between container and nitrogen effect.

**Figure 4:**
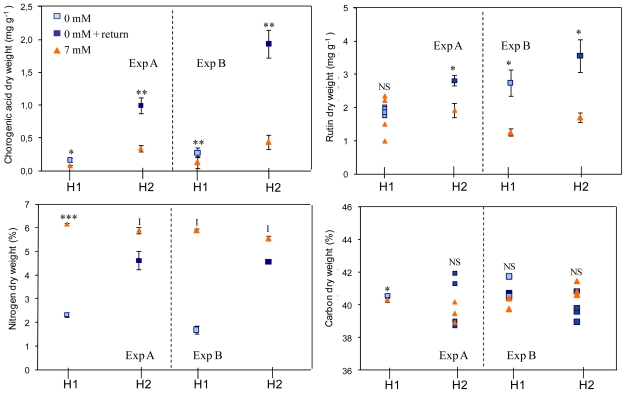
Evolution of contents in phenolic compounds chlorogenic acid and rutin in mg.g^−1^ dry weight and contents in total nitrogen and carbon in percentage of dry weight (%), during experiments A and B. Data are mean ± SE calculated on four plants. Legends are similar to Figure 3. Stars indicate significant effect of nitrogen. NS, not significant, *i.e.*, *P*>0.1; *, *P* < 0.05; **, *P* < 0.01; *** *P* < 0.001; I, interaction between container and nitrogen effect.

Firstly, sixteen plants were placed on the rich medium containing 7 mM NO_3_ ^−^ for 10 days (Figure 2). The plants were then separated into two groups of eight plants and transferred to two media: a rich medium containing 7 mM NO_3_ ^−^ (referred to hereinafter as 7mM) and a deficient medium containing no NO_3_ ^−^ (referred to as 0 mM). The plants remained on these two media for 10 days during the first experiment (Experiment A) and 19 days during the second experiment (Experiment B). At the end of this period, four plants that had been subjected to deprivation (referred as treated plant) and four control plants were harvested. The other eight plants remained under hydroponic conditions for a further five days, during which they were all grown again on a nitrogen-rich solution (7 mM) until they were harvested (in the text this period is referred to as the period of return to normal). Lovdal and coworkers [14] observed a more marked increase in flavonoids after 8 days compared to 4 days of nitrogen depletion. In order to maximize plant responses to nitrogen depletion, our plants were submitted to 10 days depletion (Experiment A) and then we doubled this time in experiment B (19 days of depletion).

### 4.2. Plant Harvesting, Sampling and Chemical Analyses

At each harvest, four plants per treatment (two containers) were collected. All fresh matter was determined by organ type: leaves, stems and roots. For chemical analyses, only the leaves were retained, frozen into liquid nitrogen and stored at − 20 °C until freeze-drying. Following this procedure, the samples were weighed to determine the dry matter (DM) and reduced to powder using a ball mill (MM200, Restch, France).

**Figure 5:**
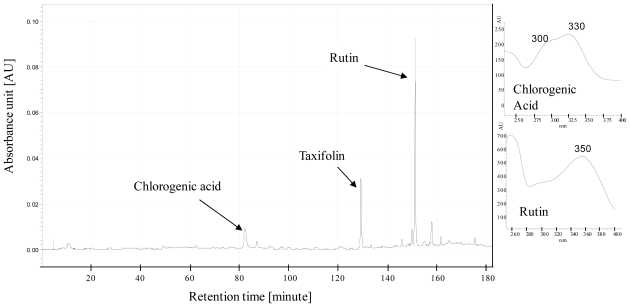
Chromatogram and UV spectra of phenolic compounds found in tomato leaves (cv Microtom). Detection was at 330 nm, Taxifolin is added as an internal standard.

The percentages of total carbon and nitrogen were obtained from 5 mg DM using gas chromatography with a carbon/nitrogen auto-analyzer (Flash EA 1112, Thermo Finnigan, USA). Polyphenols were assayed by HPLC on 50 mg DM, according to the method described in [28]. The samples were analyzed using a Beckman System Gold instrument consisting of a 508 autosampler and a dual wavelength detector (module 166), set at 330 nm for quantifications. Chromatographic separations were performed on a Lichrospher RP-18 end-capped column (4 × 250 mm, 5 μm, Merck, Darmstadt, Germany) fitted with a Lichrospher RP-18 guard column (5 μm, Merck). The mobile phase was a binary solvent system consisting of (A) water adjusted to pH 2.6 with orthophosphoric acid and (B) methanol. The gradient (from 3% to 60% of B in 180 min) was eluted at a flow rate of 0.5 mL min^−1^ at room temperature. Compounds were identified by their absorption spectra (Figure 5). All compounds were correctly separated, and quantification was based on peak surface area, referred to as a standard curve. Phenolic contents were expressed as mg per g Dry Weight (DW). Taxifolin (50 μL of a solution at 2 mg.mL^−1^ methanol, Extrasynthèse, Lyon, France) was added as an internal standard. Rutin and chlorogenic acid standards were purchased from Sigma (Sigma, Saint Quentin-Fallavier, France).

At each harvest and for each experiment we performed a two-way analysis of variance (ANOVA) using XLSTAT (Addinsoft, France) to compare control and deprived plants. The effect of nitrogen supply, the container, as a block effect, and the interaction between them were considered. The data shown on the figure are means calculated on the four plants per treatment, ± standard error (SE). When the growth conditions had a significant effect *i.e.*, container effect observed in the ANOVA analyses, the four plants per treatment were shown.

The differences obtained between the treatments were expressed as percentage variations calculated versus control plants grown on a nitrogen-rich medium throughout the study period.

## 5. Conclusions

The present work reveals part of the tomato plant strategy when submitted to a temporary nitrogen deprivation. A nitrogen deprivation period of 10 or 19 days induced a persistent effect on the phenolics content in leaves of tomato plants. Indeed we quantified a higher content of phenolics in leaves of tomato plants grown with no nitrogen in comparison to control plants grown with 7 mM NO_3_ ^−^. Moreover, the content of chlorogenic acid and rutin remained higher in plants that had previously been deprived compared to the control when all these plants were further cultivated on a nitrogen-rich medium for 5 days.

Furthermore, during these experiments, the duration of nitrogen stress (10 to 19 days) engendered a marked reduction in plant biomass production. It would therefore be interesting to test the impact of occasional nitrogen deprivation on plants that have already developed larger leaf area in order to determine whether such nitrogen stresses are sufficient to enhance the defenses of vegetative plant parts without affecting fruit yield.

## Figures and Tables

**Table 1:** Composition of nutrient solutions as a function of nitrate contents of the medium. Gram weights of compounds required to prepare 1 L of solution.

	Compound/Weight (g)	KH_2_PO_4_	K_2_SO_4_	Ca(NO_3_)_2_, 4H_2_O	CaSO_4_, 2H_2_O	MgSO_4_, 7H_2_O	EDTA-Fe, 1H_2_O	Oligoelements (mL/L)
**NO_3_^−^(mM)**	**0**	0.136	0.174	0	0.603	0.37	0.017	0.1
	**7**			0.827	0			

**Table 2:** Composition of the oligoelement solution. Gram weights of compounds required to prepare 1 L of solution.

Compound	MoO_3_	MnCl_2_, 4H_2_O	ZnSO_4_, 7H_2_O	CuSO_4_, 5H_2_O	H_3_BO_3_	FeSO_4_	Na_2_EDTA
**Weight (g)**	0.40	23.06	9.32	1.18	12.75	29.87	59.56
